# Comparing the effectiveness of different displays in enhancing illusions of self-movement (vection)

**DOI:** 10.3389/fpsyg.2015.00713

**Published:** 2015-06-01

**Authors:** Bernhard E. Riecke, Jacqueline D. Jordan

**Affiliations:** iSpace Lab, School of Interactive Arts and Technology, Simon Fraser University, Surrey, BC, Canada

**Keywords:** vection, virtual reality, self-motion illusion, display technologies, head-mounted displays, optic flow, visually induced motion sickness, self-motion simulation

## Abstract

Illusions of self-movement (vection) can be used in virtual reality (VR) and other applications to give users the embodied sensation that they are moving when physical movement is unfeasible or too costly. Whereas a large body of vection literature studied how various parameters of the presented visual stimulus affect vection, little is known how different display types might affect vection. As a step toward addressing this gap, we conducted three experiments to compare vection and usability parameters between commonly used VR displays, ranging from stereoscopic projection and 3D TV to high-end head-mounted display (HMD, NVIS SX111) and recent low-cost HMD (Oculus Rift). The last experiment also compared these two HMDs in their native full field of view (FOV) and a reduced, matched FOV of 72° × 45°. Participants moved along linear and curvilinear paths in the virtual environment, reported vection onset time, and rated vection intensity at the end of each trial. In addition, user ratings on immersion, motion sickness, vection, and overall preference were recorded retrospectively and compared between displays. Unexpectedly, there were no significant effects of display on vection measures. Reducing the FOV for the HMDs (from full to 72° × 45°) decreased vection onset latencies, but did not affect vection intensity. As predicted, curvilinear paths yielded earlier and more intense vection. Although vection has often been proposed to predict or even cause motion sickness, we observed no correlation for any of the displays studied. In conclusion, perceived self-motion and other user experience measures proved surprisingly tolerant toward changes in display type as long as the FOV was roughly matched. This suggests that display choice for vection research and VR applications can be largely based on other considerations as long as the provided FOV is sufficiently large.

## Introduction

Illusion of self-movement (vection) is a perceptual illusion that is increasingly used to investigate self-motion perception and potentially develop more effective virtual reality (VR) applications. The illusion occurs when one feels as if they are moving while stationary (see Howard and Howard, 1994, and discussion regarding vection terminology in Palmisano et al., 2015). For example, vection is often felt by one who is sitting on a motionless train while watching another train moving nearby. Vection primarily occurs with movement of the visual field (see Lappe et al., 1999), though research has shown that it may also be invoked with moving sound fields (see reviews in Riecke et al., 2009; Väljamäe, 2009), while walking on a circular treadmill (Bles and Kapteyn, 1977; Bles, 1981; Riecke et al., 2011, 2015), or with haptic stimulation (Brandt et al., 1977).

Vection research is a useful tool for theoretical investigation into the factors that may influence perception of self-motion. For example, research into vection induced by auditory stimuli has revealed that auditory cues can be sufficient to invoke the sensation of self-movement in blindfolded listeners (see Sakamoto et al., 2004), and that the source of a sound, such as whether it is the sound of a moving vehicle or an artificial sound without a real-life context, makes a difference in how strongly vection is experienced (Larsson et al., 2004). Due to its ability to invoke powerful and embodied sensations of self-movement, vection has also been discussed over the last decade as a means for improving VR and other self-motion simulations, where physical navigation is often unfeasible (see Riecke, 2011, for a review). As such, extensive research has been conducted to determine what factors may elicit, enhance, and prolong vection, both in the context of psychology research (see reviews in Dichgans and Brandt, 1978; Mergner and Becker, 1990; Warren and Wertheim, 1990; Palmisano et al., 2011) and, to a lesser degree, VR research (Riecke, 2011; Hettinger et al., 2014; Riecke and Schulte-Pelkum, 2015).

Recently, vection research employing VR has revealed that many factors influence the perception of self-motion, oftentimes through modulation of the elements within a display or virtual environment (VE). For example, a consistent, naturalistic VE has been found to induce greater vection over a scrambled one (Riecke et al., 2006), and the addition of global perspective jitter to optic flow was shown to reduce vection onset latencies and increase vection intensity (Palmisano et al., 2000, 2003). Virtual environments have also been useful for research into the role of non-visual stimuli and multimodal interaction in self-motion perception, such as how auditory cues that are spatially congruent with visual cues enhance vection over non-spatial sound (Riecke et al., 2009).

While there is a large body of research investigating how various parameters of the presented visual stimulus affect vection (see reviews in Dichgans and Brandt, 1978; Mergner and Becker, 1990; Warren and Wertheim, 1990; Palmisano et al., 2011; Riecke, 2011; Hettinger et al., 2014), relatively little work has been done upon how constituents necessary to present the moving stimulus may influence vection. One such component is the physical display itself – that is, the screen upon which the stimulus is presented. Specific parameters of these displays have been investigated in vection research; for example, research has revealed that a wider field-of-view (FOV) elicits more powerful vection over a smaller FOV (Duh et al., 2001; Zhang et al., 2010), and color contrast enhances the illusion at higher levels of saturation (Patterson and York, 2009). Yet, while there is an increasing array of different traditional and VR displays available that vary widely parameters such as size and resolution, and vection and VR research utilizes a variety of displays, the displays have yet to be directly compared in terms of their potential to elicit vection. The experiments presented in this paper are intended to help address this gap in the literature. Increasing our understanding of how different display types might affect vection can help both to improve our understanding of potential underlying factors, and provide guidance selecting suitable displays for researchers and VR applications.

The purpose of this study is to directly compare three common yet distinct VR display types [stereoscopic projection, 3D TV, and head-mounted display (HMD)] in terms of their ability to elicit vection under typical simulation settings. In addition, we compared the vection-inducing potential of two wide-FOV HMDs, a high-end HMD (NVIS SX111) and a low-end HMD (Oculus Rift DK1 which is about 1% of the cost of the high-end HMD) to assess the suitability of the low-cost HMD for vection research and VR simulations. With consideration of differing display parameters in mind (see Table 1), three experiments were conducted, investigating two displays each to determine if they differ in their ability to influence vection intensity and onset latency. Because the intention of this study was to investigate the effects of display type upon vection, rather than any singular display parameter, the inherent specifications of each display were preserved in order to best determine how each display elicits vection with the parameters under which they are naturally used, even though this necessarily introduces confounds in terms of, e.g., differences in display resolution (see Table 1). In doing so, we also hoped to glean information through qualitative user opinions regarding which display parameters participants believed may have enhanced their experience of vection.

**TABLE 1:** **The parameters of the displays used in this study**.

**Display**	**Oculus Rift DK1**	**NVIS SX 111**	**Panasonic TC-P50UT50**	**InFocus IN5500**
Display type	HMD	HMD	3D television	2-projector system
Stereoscopic presentation	Parallel	Parallel	Sequential	Parallel
Resolution per eye (horizontal × vertical)	640 × 800	1280 × 1024	1920 × 1080	1920 × 1200
Binocular FOV (horizontal × vertical)	106° × 100°	102° × 64°	72° × 40.5°	72° × 45°
Aspect ratio	1.06	1.25 or 5/4	1.78 or 16/9	1.6 or 16/10
Monocular FOV	99° × 100°	76° × 64°	–	–
Screen size (m)	–	–	1.11 × 0.62	2.45 × 1.54
Binocular overlap	92° or 93%	50° or 66%	100%	100%
Binocular overlap and monocular	72° or 100%	50° or 82%	–	–
FOV in reduced FOV (72° × 45°) condition in Experiment 3	72° × 45°	61° × 45°		
Type of VR glasses	–	–	Shutter	Polarization
Contrast ratio (according to specs)	1000:1	100:1	Dynamic	2000:1
Refresh rate (Hz)	60	60	120	60

Note that there is some debate about the precise horizontal and vertical field of view (FOV) and contrast of the Oculus Rift DK1, as the vendors do not provide any concrete numbers.

The first experiment compared a stereoscopic projection screen with a 3D television using the same horizontal FOV, the second compared an Oculus Rift HMD with the same 3D television, and the third compared the low-cost Oculus Rift with a high-end NVIS SX 111 HMD. The third experiment also included a condition where the FOV of the HMDs was reduced so it could be matched between HMDs and to Exp. 1 and 2 (72° × 45°) to see how the HMDs compared once the potential confound of FOV was eliminated. In general, increasing the stimulus size (FOV) of the moving visual stimulus enhances vection (Brandt et al., 1973; Dichgans and Brandt, 1978; for a review, see Riecke, 2011). For example, Nakamura (2008) showed that vection intensity increased linearly with the size of the moving stimulus, irrespective of stimulus eccentricity. However, much of the prior research on the influence of FOV on vection has been performed using stationary displays such as projection screens or rotating drums, but not HMDs (Kenyon and Kneller, 1993; Allison et al., 1999; Duh et al., 2001; Zhang et al., 2010). Hence, there is little known about the potential influence of an HMD’s FOV on vection. Given that providing a large FOV in HMDs is one of the key technical challenges, answering this question potentially has significant applied relevance.

Because previous studies have indicated that large screen size increases performance in some behavioral and cognitive tasks, such as 3D navigation (see Czerwinski et al., 2006, for a review), we wondered whether the large stereoscopic projection screen would provide a vection benefit over the much smaller 3D television if their physical FOV was matched. To the best of our knowledge, however, no prior research directly investigated potential effects of display size on vection, so the current study was designed as a first step toward addressing this gap. For the second experiment, we hypothesized that the Oculus Rift would enhance vection over the 3D television. This hypothesis was formed because of the larger FOV of the Oculus Rift and its ability to block off stimuli in the peripheral vision beyond the display. Stimuli in the peripheral vision have been shown to modulate vection (Brandt et al., 1973), as have unattended stimuli (Kitazaki and Sato, 2003), and the Oculus Rift’s foam barriers may prevent unattended distractors in the surrounding environment from reducing vection. Finally, it was hypothesized that the Oculus Rift would enhance vection over the SX 111 in the third experiment, as its vertical FOV was considerably larger, and its binocular overlap was larger and thus more similar to natural vision (see Table 1). Furthermore, it seemed feasible that the more visible pixilation caused by the low resolution of the Oculus Rift may also provide a benefit in vection during the second and third experiments, as previous research has shown that small, barely noticeable marks on a display can increase vection intensity and lowers onset times (Riecke et al., 2004). These results were consistent with the findings in Howard and Howard (1994), where placing stationary visual stimuli in a foreground display yielded higher vection intensity and lower onset latency than in trials without a foreground object, particularly when vection was induced by low-velocity background stimuli. Similarly, Ohmi et al. (1987) found that depth cues, such as perceived distance between the two displays, did not determine which of two displays were dominant in inducing vection, but instead which display was interpreted to be the background. From this, they proposed that the dominance of a display in inducing vection is dependent upon the display being perceived as a background stimulus. As such, it is possible that pixilation may too function as a barely noticeable static foreground stimulus, establishing the VE as a background stimulus and providing participants with a relative-motion cue, as Howard and Howard (1994) proposed as the reason for their findings.

In the first two experiments, an active navigation task employed in a previous study (Riecke and Feuereissen, 2012) was used to induce vection to allow for comparability to that study, and participants self-reported vection onset and intensity. In the third experiment, however, we switched to passive locomotion along similar trajectories, to allow for comparability to most of the vection research that used passive locomotion (similar to a watching a video, without any active steering component), as well as reduce potential confounds in the data caused by active control, or potential influences of the reduced FOV on steering/task difficulty, which could have indirectly affected vection responses.

In each experiment, a qualitative post-experimental interview was administered to gage user opinions between the two displays in regard to vection intensity, motion sickness, immersion, and overall preference. From this interview, factors that participants felt enhanced, or detracted from, their experience of vection could be seen. Due to the increasing amount of interest in the association between vection and visually induced motion sickness (Hettinger et al., 2014; Keshavarz et al., 2015), motion sickness ratings were further correlated with participants’ mean vection intensity ratings by display in order to determine if the vection intensity ratings could predict the level of motion sickness that participants experienced. Previous studies have shown an increase in both vection and motion sickness when participants assumed a forced eccentric gaze position (Diels et al., 2007), or viewpoint jitter was added to the stimulus (Palmisano et al., 2007). Furthermore, some previous research observed a correlation between vection and motion sickness (Bonato et al., 2008), so it is plausible that the occurrence and intensity of vection may be able to predict or even cause visually induced motion sickness, as discussed in more detail by Keshavarz et al. (2015). If motion sickness would indeed be causally related to vection, this would be a major hurdle for many applications depicting self-motions, ranging from large-screen movies to tele-operation, immersive gaming, and VR.

Previous research showed that linear forward vection tends to be less compelling than circular vection, whereas curvilinear forward vection (induced by moving along a path of constant curvature) matched circular vection (Riecke, 2006; Trutoiu et al., 2009; Riecke and Feuereissen, 2012). Trutoiu et al. (2009) suggested that this effect occurs because curvilinear paths preserve the structure of circular optic flow, also called lamellar flow, which induces vection more readily than linear (radially expanding) flow, because lamellar flow provides the viewer with more information regarding the path that they are traveling through. In the current study, we compared linear and curvilinear paths for different display types including both stationary (projection screen and 3D TV) displays and head-worn displays (HMD at full and reduced FOV) to investigate if there might be and interaction between path type, display type, and FOV – questions that have to the best of our knowledge not been previously investigated. While we expected curvilinear paths to produce more intense and earlier vection onset overall, it seems possible that both reducing the FOV and wearing an HMD which allows for tracked head rotations might reduce this effect.

These experiments explored how VR displays differ in their ability to modulate illusions of self-movement, and which differences in display parameters participants believed may have contributed to these differences. With knowledge of the elements that differ between each display, and from user opinions collected within the interview, this study may be used as a stepping-stone to investigate more systematically how individual or combined components of the displays may influence vection, and if factors previously found to influence vection continue to do so when they are placed in a more real-realistic applied context: a VR display, where they are combined with the other potentially vection-influencing elements that compose the display. Finally, this study will determine to what degree the type of display that is used may confound vection research conducted in VR.

## Experiment 1

Previous research suggests that screen size can enhance performance in several cognitive tasks (see Czerwinski et al., 2006). For example, Tan et al. (2003) found that an increase in screen size increases performance in 3D navigation. Might screen size provide a benefit in vection as well? To the best of our knowledge, this has not previously been answered. Here, we compared two commonly used displays differing in size, a large stereoscopic projection system and a 50-inch 3D television, to determine if they yield differences in vection intensity and onset latencies as well as user preferences and usability.

### Materials and Methods

#### Participants

Twenty-five participants, aged 20–40 (nine females) were recruited via advertisements posted on an online job-seeking board, paper advertisements posted throughout the university, and an online research participation sign-up system. They were offered either course credit or monetary compensation for their participation. All studies presented in this paper were approved by the local ethics board, conducted in accordance with the declaration of Helsinki, and participants gave written informed consent prior to participating. Two participants were excluded from data analysis, such that only data from 23 were used in the statistical analysis; one for experiencing severe motion sickness, and another for technical issues that occurred during testing. Interview data for five of the participants was lost and, as such, were excluded from the qualitative analyses.

#### Stimuli

During the experiment, participants were seated on a stationary chair in a dim room behind either the projection screen or 3D TV. Noise-canceling headphones (Audiotechnica ATH-ANC7) provided computer-generated verbal instructions and played broadband noise to exclude interfering sounds from the lab during the trials.

**FIGURE 1:**
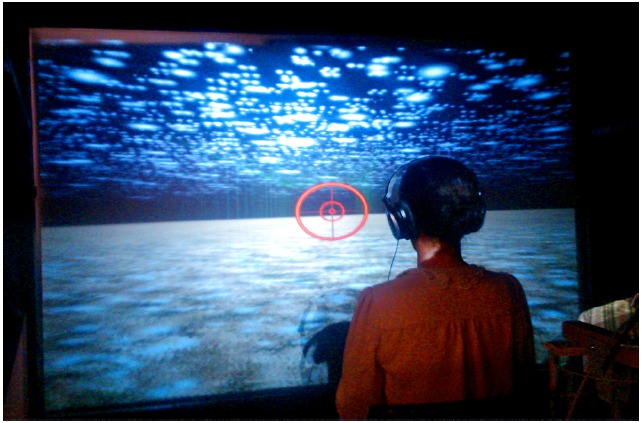
**Participant seated behind the projection screen in *Experiment 1* showing the virtual environment consisting of a grass-like ground plane and white blobs creating strong optic flow**.

A simple VE was used in order to eliminate potential confounds that could arise in a more realistic setting. The environment was generated with Worldviz Vizard software and was composed of a black background and simple grass-like ground texture, and white, snowflake-like dots provided strong optic flow with no landmarks (see Figure 1). Dynamic viewpoint-tracking was employed in both displays in order to adjust the viewing frustum to match the participant’s viewpoint with respect to the display. Using a joystick as input device, participants actively pursued an object (green cube) through the VE upon a predefined trajectory at 5 m/s. During linear trials, the object traveled in a straight line, whereas it traveled with a 24°/s turning radius during curvilinear trials. Each trial lasted for 32 s.

The stereoscopic projection system consisted of two InFocus IN5500 projectors, passive polarization glasses for parallel stereoscopic presentation, and a flat polarization-preserving screen of 2.45 m × 1.54 m. The 3D TV display consisted of a 50-inch Panasonic TC-P50UT50 3D Television with active shutter glasses for sequential stereoscopic presentation (see Table 1 for a detailed comparison of displays). The resolution of the projection system was 1920 × 1200, and 1920 × 1080 for the 3D television. For both displays, participants’ viewing distance was adjusted to yield the same horizontal FOV of 72°. The projection screen had a refresh rate of 60 Hz, while the 3D television had a refresh rate of 120 Hz as images for the left and right eye were presented sequentially using shutter glasses. Participants’ ratings of vection intensity, vection onset time, and post-experimental measures were verbalized by the participant and recorded electronically by the experimenter.

#### Experimental Design

A 2 × 2 within-subjects design was employed for this experiment. The independent variables were display type at two levels (projection screen, 3D television) and path type at two levels (linear, curvilinear). There were two direction conditions per path type (left, right). The dependent vection variables were vection onset time in seconds (self-report) collected during each trial, and post-trial ratings of vection intensity (self-report) compared to a previously established baseline. Trials were blocked by display type, and block order was counterbalanced by participant number. There were 16 trials in total, with one block per display, eight trials per block, and four trials per path type. The order of these trials was randomized within each block. The data were analyzed with one 2 × 2 repeated-measures ANOVA per dependent variable, and post-experimental ratings were compared with paired-samples *t*-tests. A linear regression was used for each display to determine if participants’ mean vection ratings could predict their motion sickness ratings.

#### Procedure

After signing an informed consent form, participants were screened for vestibular impairments with a Romberg’s balancing test (see Khasnis and Gokula, 2003, for more information regarding the test’s procedure and implications). During this task, participants put one foot in front of the other, spread their arms, and closed their eyes, for 30 s. Participants followed this procedure twice, alternating feet on the second trial. If they stumbled or fell, the participant would be unable to participate in the experiment. All participants passed this test. Following this, participants were seated within a quiet, dim room and screened for severe motion sickness and their ability to experience vection. Participants were exposed to strong vection through a task in which participants were moved passively down a curved path at a high speed for 60 s. The screening task also served as a baseline for which participants judged vection intensity in the rest of the experiment. Two baseline trials were administered per display type.

After a baseline was established, participants were instructed on how to perform the experimental task. In this task, they used a joystick to pursue a green cube through the VE until catching up with the object. Prior to beginning the experiment, participants performed one practice trial per display, which could be repeated as many times as necessary for the participant to become comfortable with the task. Following the practice trials, participants performed the main experiment, beginning with the display they were assigned to by participant number. The experiment consisted of two blocks with eight trials each, with four trials per block type and two trials per direction of the path (left, right).

During each trial, participants verbalized when they began to feel vection as soon as it occurred, and the experimenter pressed a spacebar to record the onset time. Verbal response mode was used here as participants had already the manual control task of following the green cube. Following the end of a trial, participants rated vection intensity on a 0–100% scale, using the baseline trial as the strongest intensity of vection that they could experience within the experimental context (100%), and the rating was recorded electronically by the experimenter. A break was offered between blocks, and at any time one was requested.

Following the experiment, participants were interviewed to gage their attitudes toward each display in regard to vection intensity, motion sickness, immersion, and overall preference. First, they were asked to rate how much motion sickness they experienced while using each display, on a scale from 0% (no motion sickness at all) to 100% (motion sick to the point of having to discontinue the experiment). After this, they were asked to voice their opinions as to why they thought each display invoked the level of nausea that it did, then prompted to provide their opinions about why they believed the displays may have differed in their nausea-inducing potential. After data on motion sickness were gathered, participants rated their overall vection intensity for each display on the same scale of 0% (no vection at all) to 100% (consistently as powerful as the baseline trial) and prompted for their reasoning as to what they believed may have enhanced or detracted from their experience of vection in each display. Immersion ratings were then quantified on a scale of 0% (did not feel like they were in the virtual world at all) to 100% (felt completely immersed and present within the virtual space), and participants were asked what features of the displays may have enhanced or detracted from their sense of immersion, and why they may have felt one display to be more or less immersive than the other. Finally, they chose which display they preferred and explained the reasons for this preference. In all, the experiment took about 1 h.

### Results

#### Vection Intensity

**FIGURE 2:**
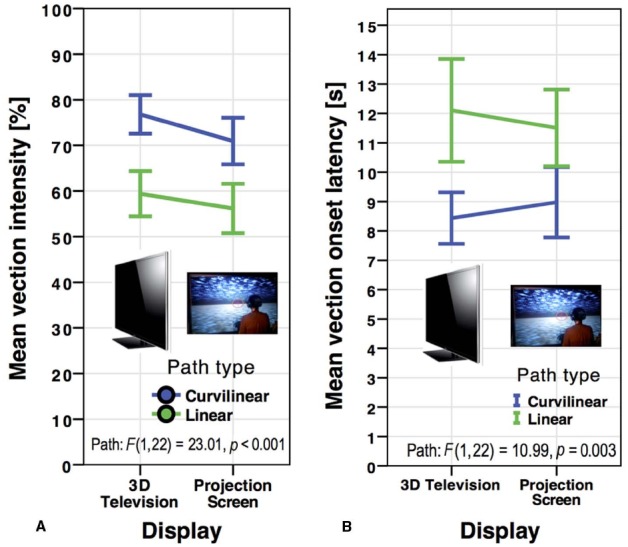
**Mean vection intensity (A) and vection onset latencies (B) for the 3D television and the projection screen by path type in *Experiment 1*.** Error bars represent standard errors of the mean.

While a trend occurred toward higher vection intensity ratings for the 3D television (*M* = 68.10, SD = 23.52) over the projection screen (*M* = 63.57, SD = 26.05), this trend did not reach significance, *F*(1,22) = 1.45, *p* = 0.241, ${\text{η}}_{p}^{2}$ = 0.062 (see Figure 2A). However, linear paths (*M* = 57.79, SD = 24.66) induced overall less intense vection than curvilinear paths (*M* = 73.88, SD = 22.41), *F*(1,22) = 23.01, *p* < 0.001, ${\text{η}}_{p}^{2}$ = 0.511. Display and path type did not interact significantly, *F*(1,22) = 0.358, *p* = 0.556, ${\text{η}}_{p}^{2}$ = 0.016.

#### Vection Onset Time

The results for vection onset latencies mirrored those of vection intensity (see Figure 2B). There was no main effect of vection onset time between the 3D television (*M* = 10.27, SD = 6.82) and the projection screen (*M* = 10.24, SD = 6.08), *F*(1,22) = 0.001, *p* = 0.970, ${\text{η}}_{p}^{2}$ < 0.001. Linear (*M* = 11.81, SD = 7.33) paths produced overall higher vection onset latencies than curvilinear (*M* = 8.71, SD = 4.98) paths, *F*(1,22) = 10.99, *p* = 0.003, ${\text{η}}_{p}^{2}$ = 0.333. There was no significant interaction between display type and path type, *F*(1,22) = 0.521, *p* = 0.478, ${\text{η}}_{p}^{2}$ = 0.023.

In summary, while curvilinear paths resulted in more intense vection and earlier vection onset than linear paths, no main effect of display arose in either vection measure.

#### Post-Experimental Interview

***Vection intensity***

There was no significant difference in post-experimental ratings of vection intensity between the projection screen (*M* = 71.61, SD = 18.86) and the 3D television (*M* = 69.50, SD = 19.49), *t*(17) = 0.518, *p* = 0.611.

***Immersion***

Although 11 of the 18 interviewed participants (qualitative data from five participants were missing) found the projection screen to be more immersive, no main effect arose in ratings of immersion between the projection screen (*M* = 63.61, SD = 20.78) and the 3D television (M = 63.06, SD = 18.72), *t*(17) = 0.131, *p* = 0.898. The most commonly given causes for a projection screen preference were the larger size of the display and the smoothness of the graphics. Participants who felt that the television was more immersive referenced the clarity and high resolution of the display – even though the actual resolution of the 3D TV was not any larger than for the projection screen.

***Motion sickness and correlation with vection***

**FIGURE 3:**
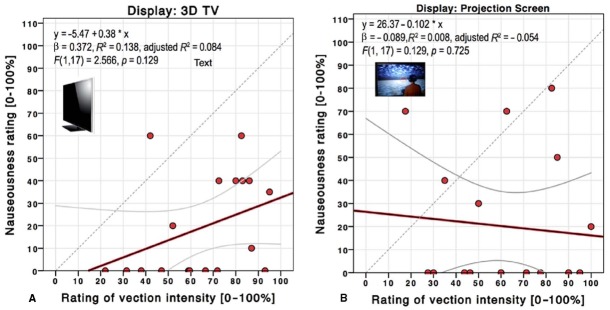
**Scatterplot and linear regression of nauseousness over vection intensity ratings for the 3D TV (A) and projection screen (B) in *Experiment 1*.** The top inset depicts the linear regression function and statistics results. Gray lines depict 95% confidence intervals. The gray dashed line depicts the diagonal for reference.

Mean ratings of motion sickness between the projection screen (*M* = 20.00, SD = 29.10) and the 3D television (*M* = 19.17, SD = 22.64) yielded no significant difference, *t*(17) = 0.196, *p* = 0.847. A linear regression was conducted to determine if participants’ vection intensity ratings could predict motion sickness ratings for each display. Vection intensity ratings did not predict motion sickness ratings for the 3D television (see Figure 3A), *R*^2^ = 0.138, *F*(1,17) = 2.566, *p* = 0.129, nor did they predict motion sickness ratings for the projection screen (see Figure 3B), *R*^2^ = 0.008, *F*(1,17) = 0.129, *p* = 0.725.

***Overall preference***

Eleven of the 18 interviewed participants preferred the projection screen. These participants felt that the projection screen had softer, more life-like graphics. Some participants mentioned that the sharpness of the 3D television caused them discomfort, such as headaches, for the close viewing distance used for this study. The eight participants who preferred the 3D television did so due to the clarity and sharpness of the image.

### Discussion

The purpose of this experiment was to determine whether there is a difference in vection intensity and onset time between a stereoscopic projection system and a 3D television. Participants’ ratings of motion sickness, immersion, and overall preference were also compared. Because screen size has been shown to increase performance in tasks such as 3D navigation (Tan et al., 2003), we wondered if the projection screen would enhance vection over the television due to its large screen size. Results revealed no main effect of display in neither vection intensity nor onset time, nor in any of the qualities rated in the post-experimental interview. That is, although many factors have been shown to influence vection onset time and intensity (see Hettinger et al., 2014 and Riecke, 2011, for reviews), the characteristics that differ between the projection screen and 3D television seem to have no quantifiable overall effect upon vection or any of the other measures used in this experiment as a parameter of a display.

There are several individual differences between the two displays that one might have expected to affect vection. For example, the 3D television possesses higher color saturation, which has been shown to enhance vection (Patterson and York, 2009). Although the size of the projection screen may be another such characteristic, the projection screen’s failure to enhance vection over the 3D television suggests that large screen size may not provide a benefit to vection, despite its helpfulness in other 3D tasks (Czerwinski et al., 2006). Alternatively, an effect yielded by differences in screen size or contrast in a highly controlled study that only manipulates the single factor of interest may diminish when placed in the context of displays, where the variables that differ between displays can be numerous and perhaps effect vection differently in combination with one another. To better understand how single elements of a display may influence vection, such as luminance, contrast, resolution, or screen size, future investigations that systematically vary one parameter at a time are needed. Here, however, the purpose was to compare two displays in a typical viewing configuration used in research and applications.

## Experiment 2

Brandt et al. (1973) reported that vection was primarily induced by stimuli in the peripheral vision. Although this notion of peripheral dominance has been disputed (Andersen and Braunstein, 1985; Howard and Heckmann, 1989; Nakamura, 2008), it is clear that peripheral vision can play a major role in inducing vection, especially if the spatial frequencies of the stimuli are low enough to match the eyes’ reduced peripheral resolution (Palmisano and Gillam, 1998).

Further research suggests that unattended stimuli can modulate vection (Kitazaki and Sato, 2003). As such, it may be possible that HMDs that block peripheral vision provide a vection benefit over displays that allow the user’s visual field to extend into the environment beyond the display. HMDs have also been found to produce greater levels of self-reported disorientation over traditional displays during a target search task (Morphew et al., 2004). Because vection has been associated with disorientation quantified through levels of postural instability (Fushiki et al., 2005), the higher levels of disorientation found in HMDs may be indicative of greater vection-inducing potential. The relationship between vection and postural sway was supported by the recent finding that individual differences in postural instability can predict vection intensity when participants view smooth radial optic flow (Palmisano et al., 2014). More specifically, there was a positive correlation between Romberg quotient, calculated by levels of spontaneous postural sway with closed eyes divided by levels of postural sway with open eyes, and the levels of vection elicited by smooth radial flow. In contrast, there was a negative correlation between Romberg quotient and vertical oscillating flow. This suggests that those who rely more heavily on vision for postural stability are more susceptible to smooth radial vection and less susceptible to vertical oscillating vection. Although this study did not explore how display type may influence disorientation or vection, it appears that there is a strong link between the two.

Research has also revealed that barely noticeable foreground marks increased vection intensity and lowered onset latencies (Riecke et al., 2004), so it is possible that the greater pixilation of the Oculus Rift may mimic this effect. This might be mediated by the pixilation providing a stationary foreground stimulus with respect to which moving stimulus might be more likely to be perceived as background motion, a factor that has repeatedly been shown to enhance vection (Ohmi et al., 1987; Howard and Heckmann, 1989; Howard and Howard, 1994; Nakamura, 2006). In addition, the native FOV of the HMD used (Oculus Rift DK1) was larger than the FOV under which the 3D TV could be comfortably viewed, which was also expected to enhance vection for the HMD (Brandt et al., 1973; Nakamura, 2008).

In sum, the purpose of *Experiment 2* was to determine if a HMD (Oculus Rift) that blocks out potential peripheral distractors, has a slightly larger FOV, and provides more pixelated stimuli than the 3D television will yield higher vection intensity and earlier onset time. To this end, we used the same experimental design and paradigm employed in *Experiment 1* apart from the differences described below.

### Materials and Methods

Twenty-three participants (11 females) were recruited through online job-posting websites and emails circulated to undergraduate courses for Experiment 2. Their ages ranged from 19 to 53. This experiment was approved by the local ethics board, and all participants gave informed consent and were awarded $15 or course credit in compensation. Two participants were excluded from the data analysis – one for providing vection intensity ratings that exceeded the 0–100 rating scale and failing to report vection onset, and another for technical issues that occurred during testing. Seventeen participants conducted the post-experimental interview. The displays compared in this experiment were an Oculus Rift DK1 HMD and the same Panasonic 3D Television used in *Experiment 1* (see Table 1 for a detailed comparison of various display parameters). Stereoscopic parallel presentation was at about 93% partial overlap in the Oculus Rift. The Oculus Rift had a resolution of 640 × 800 per eye, while the 3D television’s resolution was 1920 × 1080. Note that the native FOV of the Oculus Rift was about 106° × 100° and thus substantially larger than the FOV of 72° × 40.5° under which the 3D TV was viewed. Even though the FOV of the 3D TV could theoretically be increased by seating participants closer to the screen, pre-tests had shown that seating participants any closer would increase viewing discomfort and eye strain considerably and was thus unfeasible. To address this confound of FOV, *Experiment 3* included a reduced-FOV condition for the HMD that mimicked the FOV of the 3D TV and projection screen.

The experimental paradigm and procedure used in *Experiment 1* was also used in *Experiment 2*, but only qualitative data were collected in the post-experimental interview in order to obtain a greater depth of qualitative information regarding each domain while preserving the relatively small amount of time it took to conduct the experiment. Interview data from six participants were excluded because a second experimenter collected only quantitative ratings for these participants. As before, a 2 × 2 repeated-measures ANOVA was used to analyze the data for each dependent variable.

### Results

#### Vection Intensity

**FIGURE 4:**
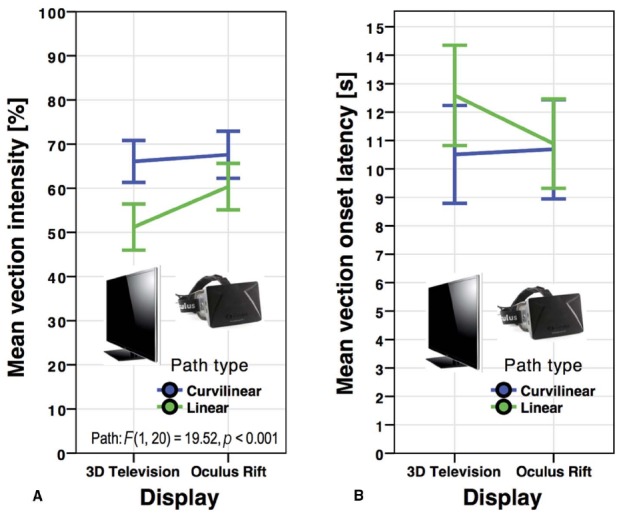
**Mean vection intensity ratings (A) and vection onset latencies (B) for the Oculus Rift and 3D television by path type in *Experiment 2*.** Error bars represent standard errors of the mean.

Although there was a trend of higher intensity ratings in the Oculus Rift HMD (*M* = 63.97, SD = 24.29) versus the 3D television (*M* = 58.63, SD = 23.79), this trend did not reach significance, *F*(1,20) = 2.731, *p* = 0.114, ${\text{η}}_{p}^{2}$ = 0.120 (see Figure 4A). Linear paths (*M* = 55.78, SD = 24.20) induced less intense vection than curvilinear (*M* = 66.82, SD = 22.85) paths, *F*(1,20) = 19.52, *p* < 0.001, ${\text{η}}_{p}^{2}$ = 0.494. No significant interaction occurred between display type and path type, *F*(1,20) = 2.42, *p* = 0.136, ${\text{η}}_{p}^{2}$ = 0.108.

#### Vection Onset Time

There was no main effect in vection onset time between the Oculus Rift (*M* = 10.79, SD = 7.51) and the 3D television (*M* = 11.55, SD = 7.99), *F*(1,20) = 1.17, *p* = 0.292, ${\text{η}}_{p}^{2}$ = 0.055 (see Figure 4B). Unlike the onset latencies in *Experiment 1*, the trend toward higher onset times in linear (*M* = 11.74, SD = 7.61) versus curvilinear (*M* = 10.60, SD = 7.84) paths did not reach significance, *F*(1,20) = 2.12, *p* = 0.161, ${\text{η}}_{p}^{2}$ = 0.096. Display type and path type did not interact significantly, *F*(1,20) = 2.17, *p* = 0.157, ${\text{η}}_{p}^{2}$ = 0.098.

In summary, no main effect or interaction occurred in either measure of vection, aside from higher ratings of vection intensity in curvilinear paths in comparison to linear paths.

#### Post-Experimental Interview

***Vection intensity***

All but three participants stated that vection intensity was greater in the HMD over the 3D television. One participant felt that it was stronger in the HMD when they moved their head from side to side. Another believed that the HMD provided stronger vection because it was “like goggles,” allowing them to focus more upon the VE than the 3D television. Those who felt that vection intensity was higher in the 3D television believed that the higher resolution of the television facilitated the illusion.

***Immersion***

Thirteen out of 17 participants who were interviewed felt that the HMD was more immersive. The reasons given for the HMD preference were that there were no outside distractions, whereas one could see beyond the 3D television in their periphery, even though the room was darkened. Two participants who preferred the 3D television mentioned that the HMD’s resolution was too blurry and pixilated, thus lessening immersion.

***Motion sickness***

Four of those who participated experienced motion sickness. Of these participants, two experienced more motion sickness with the HMD, and the other two felt more motion sickness with the 3D television. One participant remarked that the HMD’s blurriness caused the discomfort, while a participant who felt more motion sick during 3D television trials stated that the display made him feel very dizzy, particularly because he was seated so close to the screen.

***Overall preference***

Eleven of the 17 participants preferred the HMD to the 3D television. All but two participants with this preference mentioned that the HMD “blocks out the outside influences,” thus increasing immersion. Those who preferred the 3D television had more varied answers. Three participants found the HMD to be too heavy, causing discomfort, while another disliked the HMD’s head tracking. Two participants preferred the 3D television’s higher resolution.

### Discussion

The purpose of this experiment was to compare vection and user experience between a 3D television and a low-cost Oculus Rift HMD, both displays that are more commonly used in research and applications due to their increased availability and affordability. It was hypothesized that the HMD would induce more powerful vection, as it blocks out peripheral unattended stimuli that may influence vection (Brandt et al., 1973; Kitazaki and Sato, 2003), provided a larger FOV (Brandt et al., 1973, Nakamura, 2008), and has greater pixilation that may be similar to foreground marks previously shown to enhance vection (Riecke et al., 2004). The experimental results indicated no quantifiable difference in either measure of vection between the displays. That is, despite the potentially vection-reducing peripheral stimuli obscured by the Oculus Rift’s foam barriers, its larger FOV, and the evidence that pixilation might increase vection intensity, vection seems to be surprisingly tolerant toward the differences between these two displays. It is feasible that other factors in favor of the 3D television such as its higher resolution and contrast might have compensated for any potential benefits of the HMD.

Despite the absence of a difference in vection onset and intensity during the experiment, the post-experimental interview revealed that most participants felt that vection was greater in the Oculus Rift compared to the 3D television. Perhaps this retrospective measure is more robust in picking up differences in vection intensity in the context of overall user experience, though it is likely an unreliable measure of differences in vection intensity during task performance. Although most participants did not verbalize why they felt that vection was stronger with the HMD, one participant felt that the head tracking increased vection, while another referenced the HMD’s ability to block out the surrounding environment as the reason for the vection increase. Perhaps well-controlled investigations into the influence of head tracking and the presence of peripheral stationary distractors would allow for greater insight into whether these factors may influence vection during task performance, and how they influence overall user experience.

While it is puzzling that the retrospective assessment of vection differs from the experimental results, the number of participants who rated the HMD to induce greater vection was similar to the number of participants who found the HMD to be more immersive. Furthermore, the reason why most participants felt the HMD to be more immersive was similar to one of the reasons given as to why the HMD induced greater vection. Future research may investigate possible correlations between subjective ratings of immersion and vection intensity ratings. This seems conceivable, given that spatial presence in a simulated VE has been previously found to correlate with the convincingness of vection, and involvement in the VE was correlated with vection onset latencies (Riecke et al., 2006; Riecke and Schulte-Pelkum, 2015).

## Experiment 3

With the advent of affordable HMDs like the Oculus Rift or Sony Morpheus, VR is becoming more accessible. However, it is unknown as to whether these displays differ from HMDs that are more costly in domains that enhance one’s experience of the VE, such as vection and immersion. The purpose of this experiment was to compare vection intensity and onset latencies as well as qualitative measures in two HMDs: a high-end NVIS SX 111, and a low-cost Oculus Rift HMD. The influence of FOV upon these measures of vection was also analyzed. As mentioned in *Experiment 2*, the Oculus Rift has a more visible level of pixilation, which may provide a benefit to vection like that observed when a display has barely noticeable stationary foreground marks (Riecke et al., 2004). Furthermore, while the SX 111 has greater resolution, the Oculus Rift has greater luminance and stereoscopic overlap more similar to natural vision (see detailed comparison in Table 1). Recently, Allison et al. (2014) found that binocular stereopsis enhances vertical linear vection. The proposed explanation for this was that cyclopean features, which can only be seen with stereopsis, created a more compelling perception of movement in the optic flow stimulus. Because the Oculus Rift has more naturalistic stereopsis and greater pixilation, it was hypothesized that the Oculus Rift would enhance illusion of self-movement over the SX111.

### Materials and Methods

Thirty participants (11 females), aged 19–31, were recruited via email advertisement from a general elective class at Simon Fraser University. This experiment was approved by the local ethics board, and all participants gave informed consent. Participants were awarded course credit for taking part in the study. Data from five participants were excluded from the ANOVA analyses of vection onset latency for failing to follow instructions in reporting vection onset time. For the vection intensity ANOVA and correlation analysis between vection intensity and motion sickness, however, data from all 30 participants were used to increase power. The Oculus Rift HMD consisted of an 18 cm screen, with stereoscopic presentation at 93% partial overlap. Screen resolution was 640 horizontal × 800 vertical pixels per eye. With a monocular FOV of about 99° × 100°, this yields a binocular FOV of about 106° × 100°. The nVisor NVIS SX 111 HMD had a resolution of 1280 × 1024 per eye and a binocular overlap of 50° or 66%. With a monocular FOV of about 76° × 64°, this results in a combined binocular FOV of about 102° × 64°. Both displays had a refresh rate of 60 Hz. See also Table 1 for a more detailed comparison of the displays used. For the control condition, the combined binocular FOV of each HMD was adjusted to 72° × 45° to match that of the projection screen and 3D TV used in *Experiment 1* and *2*.

Although the same VE and procedure from *Experiment 1* was used for this experiment, we switched to passive locomotion along similar trajectories for this experiment, to allow for comparability to most of the vection research that used passive locomotion (similar to a watching a video, without any active steering component), as well as reduce potential confounds in the data caused by active control, or potential influences of the reduced FOV on steering/task difficulty, which could have indirectly affected vection responses.

This experiment employed a 2 × 2 × 2 repeated-measures design, with display at two levels (Oculus Rift, SX 111), path type at two levels (linear, curvilinear), and FOV at two levels (full native FOV, reduced FOV matched between HMDs). Participants took part in two blocks per display type – two trials per condition and eight total trials within each block.

### Results

#### Vection Intensity

**FIGURE 5:**
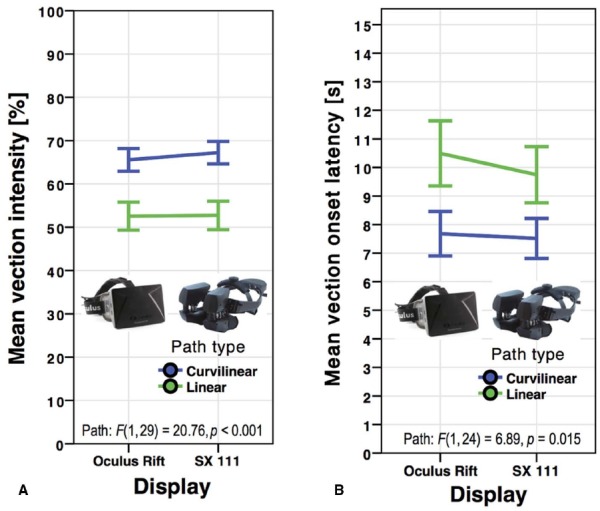
**Mean vection intensity ratings (A) and vection onset latencies (B) for the Oculus Rift and NVIS SX111 HMD by path type in *Experiment 3*.** Error bars represent standard errors of the mean.

**FIGURE 6:**
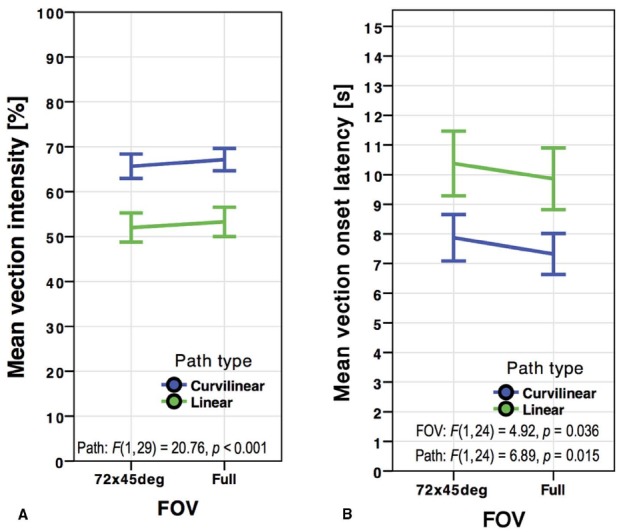
**Mean vection intensity ratings (A) and vection onset latencies (B) for the FOV (restricted versus full) by path type in *Experiment 3*.** Error bars represent standard errors of the mean.

Similar to the first two experiments, there was no significant main effect of vection intensity between the SX 111 (*M* = 59.99, SD = 24.05) and the Oculus Rift (*M* = 59.06, SD = 23.52), *F*(1,29) = 0.444, *p* = 0.510, ${\text{η}}_{p}^{2}$ = 0.015 (see Figure 5A). Furthermore, the trend toward higher intensity ratings for full FOV (*M* = 60.21, SD = 23.44) versus controlled FOV (*M* = 58.83, SD = 24.13) did not reach significance, *F*(1,29) = 2.49, *p* = 0.126, ${\text{η}}_{p}^{2}$ = 0.079 (see Figure 6A).

Linear paths (*M* = 52.64, SD = 25.15) induced overall less intense vection than curvilinear paths (*M* = 66.40, SD = 20.12), *F*(1,29) = 20.76, *p* < 0.001, ${\text{η}}_{p}^{2}$ = 0.417. No significant interaction occurred between display and FOV, *F*(1,29) = 2.90, *p* = 0.099, ${\text{η}}_{p}^{2}$ = 0.091, nor between display and path, *F*(1,29) = 0.999, *p* = 0.326, ${\text{η}}_{p}^{2}$ = 0.033. There was also no significant interaction between FOV and path type, *F*(1,29) = 0.016, *p* = 0.899, ${\text{η}}_{p}^{2}$ = 0.001, or between FOV, display type, and path, *F*(1,29) = 0.004, *p* = 0.952, ${\text{η}}_{p}^{2}$ < 0.001.

#### Vection Onset Time

No main effect of vection onset time arose between the SX 111 (*M* = 8.63, SD = 6.11) and the Oculus Rift (*M* = 9.09, SD = 7.01), *F*(1,24) = 0.761, *p* = 0.392, ${\text{η}}_{p}^{2}$ = 0.031 (see Figure 5B). However, unlike the results for vection intensity, the HMD’s native unrestricted FOV produced significantly shorter onset latencies (*M* = 8.59, SD = 6.34) than controlled FOV of 72° × 45° (*M* = 9.12, SD = 6.80), *F*(1,24) = 4.92, *p* = 0.036, ${\text{η}}_{p}^{2}$ = 0.170 (see Figure 6B). Linear (*M* = 10.12, SD = 7.50) paths produced overall higher vection onset latencies than curvilinear (*M* = 7.60, SD = 5.21) paths, *F*(1,24) = 6.89, *p* = 0.015, ${\text{η}}_{p}^{2}$ = 223.

No significant interaction occurred in onset time between display and FOV, *F*(1,24) = 1.77, *p* = 0.195, ${\text{η}}_{p}^{2}$ = 0.069, nor between display and path, *F*(1,24) = 0.502, *p* = 0.485, ${\text{η}}_{p}^{2}$ = 0.020. There was also no significant interaction between FOV and path type, *F*(1,24) = 0.222, *p* = 0.642, ${\text{η}}_{p}^{2}$ = 0.009, or between FOV, display type, and path, *F*(1,24) = 0.042, *p* = 0.838, ${\text{η}}_{p}^{2}$ = 0.002.

To summarize these results, no main effect of display was revealed in either measure of vection, while curvilinear paths produced significantly higher vection intensity ratings and lower onset latencies. Although the trend toward higher vection intensity ratings in the full FOV condition did not reach significance, onset latencies were significantly reduced when the displays were used at full FOV.

#### Post-Experimental Interview

***Vection intensity***

Although vection intensity rating of the SX 111 (*M* = 61.08, SD = 21.46) were slightly higher compared to the Oculus Rift (*M* = 58.72, SD = 21.48), this trend did not reach significance, *t*(24) = 0.915, *p* = 0.369. Some participants remarked that the higher resolution of the SX 111 contributed to their experience of vection, while others noted that the discomfort caused by the heaviness of the display may have reduced it. One participant remarked that the lower resolution of the Oculus Rift may have enhanced their experience of vection.

***Immersion***

No significant difference occurred between mean immersion ratings for the Oculus Rift (*M* = 60.64, SD = 23.85) and the SX 111 (*M* = 59.68, SD = 23.67), *t*(24) = 0.333, *p* = 0.742. Participants who rated the SX 111 to be more immersive referenced the high resolution and clarity of the graphics, while those who found the Oculus Rift more immersive noted the comfort and lightness of the display. Those who found the Oculus Rift to be more immersive also felt that the image in the SX 111 seemed smaller, reducing their presence within the VE.

***Motion sickness and correlation with vection***

**FIGURE 7:**
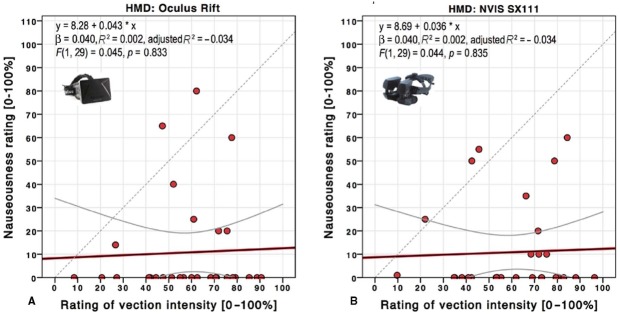
**Scatterplot and linear regression of nauseousness over vection intensity ratings for the Oculus Rift (A) and NVIS SX 111 HMD (B) in *Experiment 3*.** The top inset depicts the linear regression function and statistics results. Gray lines depict 95% confidence intervals. The gray dashed line depicts the diagonal for reference.

**FIGURE 8:**
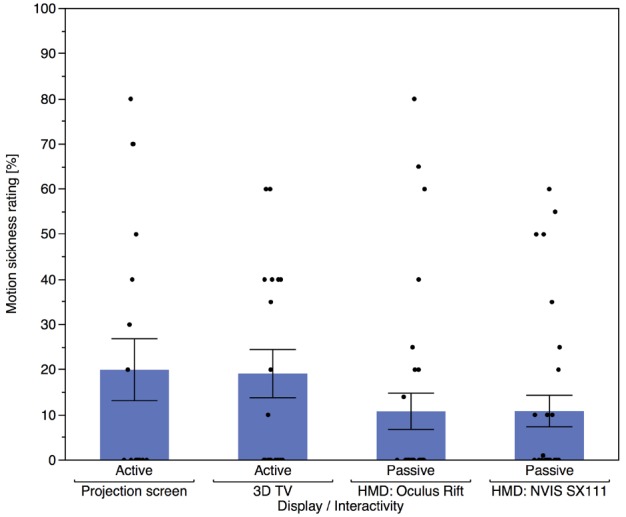
**Mean nauseousness ratings per display in *Experiment 1* and *3*.** Error bars depict standard errors of the mean. Participants’ individual responses are depicted as black dots.

Very few participants experienced any motion sickness during this experiment. There was no significant difference in ratings of motion sickness between the SX 111 (*M* = 8.84, SD = 16.71) and the Oculus Rift (*M* = 7.96, SD = 18.30), *t*(24) = 0.287, *p* = 0.777. Linear regressions were performed to determine if participants’ mean vection intensity can predict motion sickness ratings for each display. Mean vection intensity did not predict motion sickness ratings, neither for the Oculus Rift [see Figure 7A, *R*^2^ = 0.002, *F*(1,28) = 0.045, *p* = 0.834] nor for the SX 111 [see Figure 7B, *R*^2^ = 0.002, *F*(1,28) = 0.045, *p* = 0.836]. As illustrated in Figure 8, motion sickness ratings differed considerably between participants, and there was a trend toward reduced motion sickness for the passive locomotion using different HMDs in *Experiment 3* compared to the active locomotion using a projection screen or 3D TV in *Experiment 1*.

***Overall preference***

Nine of the 25 interviewed participants preferred the SX 111. All of these participants preferred this display due to the high resolution of the graphics. Those who preferred the Oculus Rift did so due to the relative comfort and lightness of the display, which some remarked contributed to their experiences of vection and immersion. One participant remarked that the higher luminance of the Oculus Rift was preferable, while another noted that the lower resolution of the display made the environment seem more realistic.

### Discussion

The purpose of this experiment to compare vection and user experience between a consumer-accessible Oculus Rift HMD and an expensive SX 111 HMD at their full FOV, and when the FOV was reduced to be equal between the two HMDs and to match the 3D TV and projection screen displays used in *Experiment 1 and 2*. From the results, it can be concluded that vection is again tolerant toward differences in display, despite the number of parameters that differ between the two displays. Furthermore, despite previous research (Duh et al., 2001; Zhang et al., 2010) uncovering a robust vection-facilitating effect of increasing the FOV, the effect in this experiment was only present for vection onset latency.

Previous research has found that presenting the VE with a wide horizontal FOV increases illusions of self-motion. However, these studies have been conducted upon displays that are not worn by the observer, such as a projection screen (Kenyon and Kneller, 1993) and a rotating drum (Brandt et al., 1973; Zhang et al., 2010). Perhaps there is a quality to HMDs that reduces the effect that FOV has upon vection to the degree uncovered in this experiment. Future research could investigate the interaction between FOV and display between an HMD and a different type of display, such as a projection screen, in order to determine if FOV does indeed have a comparatively smaller influence on vection in HMDs. Although no interaction was found between path type and FOV in this experiment, it may be possible that FOV plays a smaller part in the experience of linear and curvilinear vection when compared to, for example, roll or yaw circular vection without any linear motion component (Brandt et al., 1973; Kenyon and Kneller, 1993; Allison et al., 1999; Duh et al., 2001; Nakamura, 2008; Zhang et al., 2010).

Although there was no main effect of vection intensity or onset time between the Oculus Rift and SX 111, it is plausible that a benefit to vection provided from any one parameter of the displays disappeared when combined with the other differing parameters. For example, the more naturalistic binocular overlap in the Oculus Rift should have enhanced vection over the lower binocular overlap found in the SX 111, given that higher levels of binocular stereopsis have been shown to enhance vection (Allison et al., 2014). Perhaps, when placed in the context of the many factors that compose a display, the benefit of stereopsis diminishes.

Some participants remarked that the high resolution of the SX 111 allowed them a greater experience of vection while using the display, while others believed that vection was more powerful in the Oculus Rift because the screen seemed closer to their eyes. Luminance values and comfort levels may also contribute to the experience of vection and immersion. Because user opinions were quite evenly divided between the HMDs in every category of the interview, including vection, it may be possible that individual differences, such as levels of video game and computer usage, may influence what users consider immersive, and perhaps how factors of the display influence retrospective evaluations of vection.

## General Discussion

The purpose of these studies was to determine how the type of commonly used VR displays might affect vection and user experience. Three experiments were conducted, comparing two displays each – a 3D television versus a stereoscopic projection screen (*Experiment 1*), a consumer-level Oculus Rift HMD versus a 3D television (*Experiment 2*), and a high-end NVIS SX 111 HMD versus an Oculus Rift HMD (*Experiment 3*). In *Experiment 3*, these measures of vection were further compared at the two displays’ full FOV, and when the FOV was reduced to match each other and the displays used in the prior experiments. Participants’ ratings and opinions on vection, motion sickness, immersion, and overall preference were contrasted in post-experimental interviews. Results revealed no significant main effect of the display in neither vection onset time nor vection intensity. In *Experiment 3,* a vection-facilitating effect of increasing the FOV was present only in vection onset time, despite previous research uncovering a robust effect of FOV upon vection (Brandt et al., 1973; Duh et al., 2001; Nakamura, 2008; Zhang et al., 2010). Additionally, in *Experiment 1* and *Experiment 3* there was no significant difference between the displays in post-experimental quantitative user ratings, though the qualitative data gathered in *Experiment 2* indicates a preference toward the Oculus Rift over the 3D TV in regards to retrospective assessments of vection intensity, immersion, and overall preference. The consistent vection-facilitating effect of curvilinear over linear paths observed in the current studies confirms prior research (Riecke, 2006; Trutoiu et al., 2009; Riecke and Feuereissen, 2012) and generalizes it to a larger range of display types and sizes as well as both passive and active locomotion. Note that display type did not interact with path type or FOV. That is, the vection-facilitating effect of path curvature was overall comparable amongst displays ranging from head-worn (HMD) to stationary (projection screen and 3D TV) displays and did not change with the FOV reduction in *Experiment 3*.

While previous VR research has explored how various factors, such as naturalistic visual stimuli (Riecke et al., 2006), may increase vection, it can be concluded that some of the vection-enhancing effects that have been previously observed in highly controlled paradigms disappear when combined and tested under typical viewing conditions of different VR displays. For example, the 3D television used in *Experiments 1 and 2* possesses higher color saturation, which has been shown to enhance vection (Patterson and York, 2009), while the Oculus Rift prevents users from viewing peripheral unattended stimuli that may influence vection (Brandt et al., 1973; Kitazaki and Sato, 2003) and has a high level of binocular overlap, another factor that has been shown to enhance vection (Allison et al., 2014).

Studies in other areas of brain and behavioral research have indicated that effects found while varying single factors may not generalize to more naturalistic contexts. For example, a visual search task that varied multiple qualities of the visual stimuli at a time found no effect of luminance, a property that has been shown to capture attention in more highly controlled paradigms (Kunar and Watson, 2011). It may be possible that, as vection-modulating factors increase within a context, the effects that have been observed by varying a single factor may be reduced or diminished entirely. Contrary to most experiments, the primary independent variable investigated in this experiment (display type) was composed of numerous differing parameters, and, as such, was more representative of the natural environment. Despite the presence of these differences, vection was tolerant toward changes in display type. The question arises as to whether the disappearance of previously discovered effects is restricted to the context of display, or if this may also be observed in other complex, naturalistic contexts. In the future, carefully controlled research that systematically varies the parameters of the elements within a display, such as luminance and resolution, may be used to investigate how the effects of a single display factor differ when integrated into an environment with other factors that may also influence vection.

User opinions from the post-experimental interview suggest that individual preferences may play a factor in the experience of vection. In *Experiment 3*, participants remarked that the high resolution of the SX111 allowed them a greater experience of vection while using the display, while others remarked that vection was more powerful in the Oculus Rift because the screen appeared closer to their eyes. In *Experiment 2*, some participants found that the softer, more naturalistic graphics of the projection screen increased vection, while others felt that the perceived sharpness of 3D television enhanced the illusion. It may be possible that individual preferences had an influence upon retrospective evaluations of vection.

Vection intensity ratings did not predict levels of motion sickness in *Experiment 1* and *Experiment 3*, a result consistent with some previous studies in which no correlation was found (see Webb and Griffin, 2003; Keshavarz and Hecht, 2011). Although it has been postulated that vection is a necessary prerequisite for simulator sickness (Hettinger et al., 1990), there seems to be conflicting evidence regarding whether or not there is a positive correlation between levels of vection and motion sickness. Perhaps the positive correlations found in some studies (Diels et al., 2007; Palmisano et al., 2007; Bonato et al., 2008) were the result of experimental conditions that modulate both vection and motion sickness, rather than stronger vection leading to an elevation in motion sickness. Further research into the potential influence of vection upon motion sickness is needed to clarify their relationship, as discussed in more detail in Keshavarz et al. (2014, 2015).

Because vection onset time was self-reported, the accuracy of the mean recorded time may have been reduced by trials where participants forgot to record onset time until after they had begun to experience vection. In future experiments, splitting the paradigm into two separate tasks, self-report and intensity rating, may reduce this. While this experiment was restricted to four different and relatively new displays, it is possible that these results may not generalize to other displays, particularly if their specs, such as resolution and refresh rate, differ dramatically, or if they are not based on digital presentation (e.g., optokinetic drums). Similarly, while vection was surprisingly tolerant toward changes in display, an alternative explanation may be that the different factors that compose a display counterbalance one another to remove any benefit a single factor may provide. Future research into how vection-modulating factors influence vection when combined would more definitively shed light onto how these results might be related to counterbalancing of individual factors.

In conclusion, the current study suggests that overall user experience and reported vection seems relatively tolerant toward changes in display type. Furthermore, vection and motion sickness do not seem to correlate in this experimental paradigm. Carefully planned research that varies display factors in a controlled manner to reduce potential confounds is needed, though, to more systematically investigate how of the influence of single display parameters on vection may change when other vection-enhancing parameters are included.

### Conflict of Interest Statement

The authors declare that the research was conducted in the absence of any commercial or financial relationships that could be construed as a potential conflict of interest.
